# Considerations on the Analysis of E-900 Food Additive: An NMR Perspective

**DOI:** 10.3390/foods11030297

**Published:** 2022-01-22

**Authors:** Héctor Palacios-Jordan, Anna Jané-Brunet, Eduard Jané-Brunet, Francesc Puiggròs, Núria Canela, Miguel A. Rodríguez

**Affiliations:** 1 Eurecat, Centre Tecnològic de Catalunya, Centre for Omic Sciences (COS), Joint Unit URV-EURECAT, Unique Scientific and Technical Infrastructures (ICTS), 43204 Reus, Spain; hector.palacios@eurecat.org (H.P.-J.); nuria.canela@eurecat.org (N.C.); 2 LLUIS JANE BUSQUETS (LJB) Analysis Laboratory S.L., Sant Quirze del Vallés, 08192 Barcelona, Spain; lluis.jane@ljblab.com (A.J.-B.); ljb@ljblab.com (E.J.-B.); 3 Eurecat, Centre Tecnològic de Catalunya, Biotechnology Area, 43204 Reus, Spain; francesc.puiggros@eurecat.org

**Keywords:** food additives, food analysis, food control, antifoaming agents, E900, nuclear magnetic resonance (NMR)

## Abstract

Food additives are in widespread use in the food industry to extend the shelf life of food, improve its organoleptic characteristics or facilitate industrial processing. Their use is not without controversy, which makes regulation and control crucial for food safety and public health. Among food additives, silicone-based antifoaming agents (polysiloxanes or E900) are difficult to analyze and quantify due to their polymeric nature. Currently, there is no official method of quantifying this additive in foods. In this context, nuclear magnetic resonance (NMR) is a quantitative method for speciation analysis of silicon compounds almost without known interferents. In this work, we describe the evolution of the regulation of the E900 additive, discuss different analytic methods quantifying polydimethylsiloxanes (PDMS), and propose a new method based on NMR suitable for analyzing the content of E900 in the form of PDMS in various types of food from dietary oils to marmalades and jellies, among others. The proposed method consists of a previous quantitative concentration of PDMS by liquid–liquid extraction and the monitoring of the quantification using a bis(trimethylsilyl)benzene (BTMSB) standard to control the variability, ranging within 2–7%, depending on the food. This simple, direct, and reproducible procedure for aqueous and lipidic foods may help to monitor and fill a gap in regulatory legislation regarding the E900 additive.

## 1. Food Additives

Additives are external chemical substances used during the preparation of food products in order to extend their shelf life, improve their organoleptic characteristics, or facilitate industrial processing [1]. 

The worldwide changes in dietary habits over recent years towards foods of easy preparation with pleasing flavor, high-energy density, and low cost instead of natural and unprocessed raw meals have increased the use of and demand for chemical additives in processed foods [2].

Food chemical additives were defined by the Joint World Health Organization/Food and Agriculture Organization (WHO/FAO) Committee in 1955 [3] as substances “*which are added intentionally to food, generally in small quantities, to improve its appearance, flavor, texture, or storage properties*”. In addition, substances added during the technological processing of food or to increase its nutritional value are also considered food additives. However, contaminants and other substances accidentally added to food are not included within this definition [4].

Food additives can be classified into different types according to their function, for example, to improve the sensory quality of the food (such as colorants, flavorings, texturizers, bleaching, or maturing agents), to extend its useful life as preservatives (antioxidants, antimicrobial, and even antibiotics), or to add nutritional properties (vitamins, amino acids, and essential fatty acids, among others) [5].

As these chemicals are intentionally added to food, it is essential to understand their properties, so as to ensure their adequate and safe use [6]. The importance of technological additives in food production is evident and increasing over time. However, it is necessary to be aware of the possible health burden caused by frequent exposure to large quantities of these substances [7]. The increased demand for additives in food due to changes in food consumption (type of food and patterns of food behaviour) also are met with increased consumer awareness (nutritionally, environmentally) and scrutiny of these additives. The assessment of food additives worldwide is supported by the control system of the acceptable daily intake (ADI), which determines, with a high safety margin, the maximum amount of additive that can be ingested without associated health problems. The ADI value system was developed by the Joint FAO of the United Nations and WHO Expert Committee on Food Additives [8], and it is widely used in several studies [9,10].

For all these reasons and regulatory issues, which are discussed below, the safety of all food additives is continuously monitored and quantified by regulatory agencies, and their use must always be closely controlled [11]. Despite this tight control, its addition to foods is never without controversy [12].

Among food additives, silicone-based defoamers are of particular interest due to their non-nutritional role as adjuvants in food processing and its variable polymeric chemical nature [13]. Therefore, in the food-processing industry, polydimethylsiloxanes (PDMS), categorized as E900, can be found as contaminants from packaging material [14] or intentionally added as foam-suppressing and antifoaming agents [15,16,17]. 

For this reason, as expected for any added chemical substance, food and medicinal agencies, such as the US Food and Drug Administration (FDA) or the European Food Safety Authority (EFSA), require that adding silicones in a production process should be controlled.

## 2. Chemical Characterization of Silicon Compounds Food Additives

PDMS belongs to the family of silicon compounds, but it is not the only form of silicon that can be found in foodstuffs. In foodstuffs, organic and inorganic silicon compounds may be present as a natural component, a purposeful additive, or contaminant [18].

As inorganic silicon compounds present in foodstuffs, we can find mainly derivatives of orthosilicic acid (Si(OH)_4_), such as sodium, calcium, and magnesium silicates and a hydrated silica (SiO_2_·nH_2_O). Organic silicon species (siloxanes), on the other hand, are described in food as a variety of structures of linear, branched, cyclic, and cross-linked polymers.

European Union Member States allow seven inorganic (i.e., E551, E552, E553a, E553b, E554, E556, and E559 [19,20,21]) and one organic (i.e., polydimethylsiloxane E900) silicon compounds to be used as food additives. In addition, food processing is allowed to use diatomaceous earth and/or silica earth, which have not been assigned an identification E code. 

The use of diverse silicon additives with a wide range of technical utilizations, properties and biological behavior means that there are different silicon species in food in the form of different chemical structures. This variety of silicon chemicals species highlights the importance of the use of specific and/or selective analytical methods, or at least the analytical separation or speciation of the different silicon compounds prior to analysis [22]. Major challenges in the analysis of siloxanes are due to the presence of a variety of silicon compounds that could give off analytical artifacts or a cross-response and due to possible contaminations, since they are widely applied in many consumer and personal care, technological, and industrial products [23]. 

**Figure 1:**
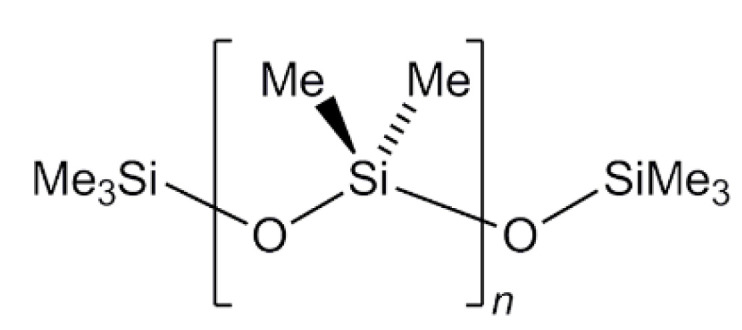
General structure of linear PDMS.

Dimethylpolysiloxane is a mixture of fully methylated linear siloxane polymers containing repeating units of the formula (CH_3_)_2_SiO and terminated with trimethylsiloxy end-blocking units ((CH_3_)_3_SiO) (Figure 1) [24]. Additional restrictions and specifications of molecular weight; end-blocking units (hydroxyl –OH, alkyls, etc.); presence or absence of linear, branched, or cyclic structures and their physical properties (such as viscosity, migration, and thermal stability) are imposed according to regulatory agencies and industry.

Among these silicon additives or contaminants, PDMS (E900) has attracted much attention, and it is in widespread use in the pharmaceutical, medicinal, and food industries due to its useful properties and being almost biologically inert [25]. However, there is an increasing public consciousness of the potential damaging consequences of continuous direct exposure to siloxanes in general [26].

Among all siloxanes, PDMS is difficult to analyze and quantify due to its polymeric nature. Therefore, the present work focused on the evaluation and control of E900 performed by regulatory agencies and the impact of its analysis and assessment specifically as a food additive.

Dimethylpolysiloxanes are mainly produced in the industry from sand treated at high temperatures [27]. This processed inorganic silicon powder could also be added as an antifoaming agent, but its insolubility and difficult dispersion in food prevents its widespread use. Thus, the silicon powder is allowed to react with chloroform (CH_3_Cl) at high temperature and pressure in the presence of copper as a catalyst to generate methylchlorosilanes. The main fraction of dimethylchlorosilane is distilled and hydrolyzed giving a mixture of cyclic dimethylpolysiloxanes (with 3–6 Si–O repeating units) and linear chains (with 30–50 Si–O repeating units) of different proportions depending on reaction conditions [28]. Elongation of linear polymers could be continued under acidic conditions until the desired chain length and viscosity is obtained [29].

Due to the production process, copper impurities or intermediates, such as chlorosilanes, silanols, or cyclic PDMS, can be found in commercial E900. In this way, and as we will see in the next section, in addition to regulating the presence and quantity of this additive in foods, the European Commission recommended setting the lower limits for toxic elements (copper, arsenic, lead, and mercury), including the range of weight-average molecular weight (Mw) and number-average molecular weight (Mn) as well as the maximum limit of cyclopolysiloxanes, silanols, and chlorosilanes in the EU specification for the E900 additive [17].

Despite these drawbacks, PDMS as a food additive has a high antifoaming activity (for the manufacturing of many foodstuffs such as wine, juice, beer, and soft drinks), good anti-adhesive properties (preventing the product or packaging from sticking), and a protective effect against thermo-oxidative processes [30]. Dimethylpolysiloxanes may depolymerize at temperatures above approximately 300 °C and under strong acidic or basic conditions, but such reactions have not usually been observed and are not expected to be observed in fried foods in oils that contain PDMS [31]. Therefore, this additive differs from the rest due to its hydrophobic nature, because it has a non-polar structure [32]. Moreover, it has low chemical and biological reactivity and is stable at high temperatures, although continued wet heat, such as saponification reactions, can cause depolymerization [17].

Due to PDMS industrial relevance, this review explores the gap in the quantification and analysis of silicone compounds, by first looking at its regulation and legislation history, then its biological and toxicological effects, and finally focusing on E900 analysis in foods.

## 3. PDMS Regulation and Legislation

Since the beginning of its production and regulation, the additive E900 has been varying its permitted levels due to the increase in knowledge of its effects on health and the environment. Many studies have been carried out in animals and humans and, consequently, its levels of exposure and consumption have been adjusted according to health and food consumption patterns.

PDMS was first evaluated as a food additive by the Joint FAO/WHO Expert Committee on Food Additives (JEFCA) in 1969 and again in 1974. Then, an acceptable daily intake (ADI) of 1.5 mg/kg of body weight (bw) was established based on a long-term toxicity study on rats performed in 1959, where no adverse effects were observed at exposition levels of 150 mg/kg bw per day [33].

In 2008, the substance was placed back on the JECFA agenda, and the previously established ADI of 0–1.5 mg/kg of body weight was withdrawn, since a new study evaluated by the Committee showed E900 effects on the corneas of the tested animals. Based on this study, an additional safety factor of 2 was included to establish a temporary ADI of 0–0.8 mg/kg body weight [15].

In 2011, JECFA considered new studies and concluded that the ocular lesions were caused by local toxicity when the eyes of laboratory animals were exposed by contact to dimethylpolysiloxane, which would be present in feed or feces, or even through grooming of contaminated fur. Therefore, the Committee re-established an ADI of 0–1.5 mg/kg bw per day [16].

In addition to this increase in the ADI from 0.8 to 1.5 mg/kg bw, the French Agency for Food, Environmental, and Occupational Health Safety (ANSES) re-evaluated the calculated exposure of PDMS based on updated human food intake patterns even to a lower value. This study, published a series of opinions on the use of various antifoaming agents as processing aids, including dimethylpolysiloxanes [34], and emphasized that the previous database was extremely limited quantitatively and qualitatively. Therefore, updating values of human intake, due to the low use levels as a processing aid, exposure levels were determined to be low (from 0.2% to 22% percent of an ADI of 1.5 mg/kg bw per day), and the ANSES concluded there was no safety concern.

Currently, the data on food consumption used to estimate the diet’s exposure to dimethylpolysiloxanes are from the EFSA Comprehensive European Food Consumption database [35].

As a rule of thumb, the amounts of PDMS in foodstuffs according to the existing regulations [17] should not exceed, in general, of 10 mg/kg of the final product with concentrations ranging from 110 to 5 mg/kg depending on the food. These levels ensure an acceptable daily intake (ADI) below 1.5 mg/kg body weight, as it was established by the international Joint Expert Committee on Food Additives (JEFCA) [16]. This amount was limited with by the assumption of the standard diets studied in different countries and the fact that silicones do not biodegrade in living organisms and are not absorbed in the digestive tract [36].

Finally, in 2020, the European Union Panel on Food Additives and Flavourings (FAF) of the EFSA provided an extensive re-evaluation of PDMS as a food additive and recommended what specifications should be updated to better describe the material used as the food additive E900 to ensure its safety of use for EU countries [17].

In doing so, the current regulation by the EFSA Panel established an ADI of 17 mg/kg bw per day for dimethylpolysiloxane (E900) and withdrew the previous value of 1.5 mg/kg bw per day, established by its Scientific Committee of Food SCF in 1990 in accordance with the JEFCA value of 1974.

However, together with this increase in the permitted level of E900, the Panel recommended to the European Commission that PDMS used as food additive should include additional information and chemicals restrictions. This information must include the range of the weight-average molecular weight (Mw) and number-average molecular weight (Mn) of the manufactured polymer used; the maximum amount of cyclopolysiloxanes and low molecular molecules of PDMS present in the mixture; assess the values of toxic metallic elements such as copper, (used as a catalyst in the industrial production) arsenic, lead, or mercury.

Following these guidelines, the name of dimethylpolysiloxanes was changed to the more accurate “poly(dimethylsiloxane)” (PDMS), and the authorized use of the substance is limited to linear polymers (without cyclic or branched structures) with molecular weights above 6.8 kDa and low levels or better total absence of impurities of toxic elements. With these restrictions, EFSA wants to avoid any absorption and adverse biological effects of PDMS, which are more associated with low-molecular-weight polymers, cyclic structures, and the presence of heavy metallic elements or impurities.

## 4. Biological and Toxicological Effects of PDMS

The suitable physico-chemical characteristics of polysiloxanes, in general, particularly PDMS, have provided many useful applications in cosmetics and health care, pharmaceutical products, medical devices, and food technology. In 2019, global sales of these compounds were 6.75 million tons. Therefore, there are some recent warnings indicating that siloxanes, especially in the form of cyclic or low-molecular-weight molecules, are becoming an uncontrollable source of pollution in the environment [37].

Dimethylpolysiloxanes are also used in cosmetic products as an excipient in pharmaceutical products and as a processing additive in food. The quantification of exposure via all these sources is not known and was therefore not considered in this review. However, it is known that today, human exposure to siloxanes through the environment [38], by cosmetic products [39], and medical devices [40] far exceeds that due to the additive E900 from food intake.

As a food additive, PDMS is generally well tolerated by humans, because its use is limited to a high-molecular-weight polymer with low impurities. The possible carcinogenic or toxic effect of changes on protein conformation; alterations in endocrine; reproductive or immune systems; and intraocular, nasolacrimal duct, or respiratory tract irritations are limited to low-weight silicon molecules [41].

In several pharmacokinetics studies on oral administration to mice, rats, monkeys, and humans, it was shown that PDMS was only absorbed to a very limited extent in the gastrointestinal tract. More than 99.9% of the orally ingested PDMS was excreted unchanged in the feces. Only low-molecular-weight cyclosiloxanes were absorbed from the gastrointestinal tract, highlighting the importance of ensuring the absence of this class of siloxanes in E900 additive [42].

Thus, high-molecular-weight PDMS is not absorbed during digestion. In addition, several ADMET, short-term and long-term toxicity and carcinogenic studies on PDMS as a food additive have been conducted in mice, rats, rabbits, dogs, monkey, as well as in humans with different results [17].

A pharmacokinetic and toxicological study highlighting the toxicity of PDMS was developed by Lukasiak and co-workers. They examined the absorption and distribution of dimethylpolysiloxane oil of low viscosity (300 centistokes) in male Wistar rats [43]. The accumulation and toxic effects of siloxanes were thoroughly studied in the blood, brain, kidneys, liver, and spleen in the animals killed after 12 days. Lukasiak and co-workers established that PDMS was preferentially absorbed by the brain and kidneys; cyclic PDMS remained in the circulatory system and partly in the kidneys. However, the internal organs showed no pathological changes attributable to siloxanes, and in 2020, the EFSA re-evaluation considered the study “as not reliable” [17].

On the other hand, Kawabe et al. [44] developed a 26 month toxicity and carcinogenic study in female and male rats with high doses of PDMS KS66 resin (up to 2 g/kg bw) without any adverse effect observed. They concluded that most of the dimethylpolysiloxane was not absorbed and excreted via feces.

Based on this study, the EFSA Panel on Food Additives and Flavorings (FAF) considered that oral exposure to dimethylpolysiloxane did not result in any systemic adverse effects in any species and at any dose tested and, thus, derived for E900 an ADI of 17 mg/kg bw in 2020 [17].

A recent study by Romano and co-workers [45] assessed the cytotoxic effect of low-molecular-weight components and conventional silicone oils with different degree of purification using in vitro cytotoxicity tests with BALB 3T3 mouse cells and human retinal pigment epithelial cells (ARPE-19). They demonstrated the absence of the cytotoxicity of silicone oils, regardless of the degree of purification.

Currently, the consensus on the use of E900 as a food additive is that it is safe; however, studies should continue to confirm this safety and degree of safety with certainty Legislation promoted by regulatory agencies, both with respect to the maximum levels allowed in food and the maximum allowed intake, efficiently avoid any problem due to PDMS intake’s biological effect. Presently, human exposure to siloxanes of all chemical nature, such as the most reactive low-molecular-weight PDMS and cyclosiloxanes, comes mainly from cosmetic and pharmaceutical products or environmental pollution, instead of E900 food additive [26].

## 5. PDMS Analysis in Food

As reported above, food and medicinal agencies, such as the FDA or the EFSA, require the control of the addition of silicones in a food product.

Currently, in the literature, there are many robust, general, and validated methods for the determination of PDMS in foodstuffs [23]. However, there is no specific legislation concerning official methods for this analysis. The choice of method depends on the laboratory analysis and, hence, there is no possibility of officially proving the criteria of the regulatory agencies. Moreover, the food matrix also determines the optimal analytical procedures to obtain suitable and reproducible results [46,47].

For example, in the case of PDMS added to edible oils and fats, an additional problem is to separate the siloxanes from the excess fat in food extracts, since they have a similar polarity. This scenario explains the wide variety and large number of analytical methods for silicon determination published in the bibliography [23,48,49,50].

For total silicon measurement, atomic spectroscopic methods with different kinds of ionization and detection analyzers, such as atomic absorption/emission spectroscopy (AAS/AES), inductively coupled plasma mass spectroscopy (ICP-MS) or optical emission spectroscopy (ICP-OES), are widely used for the determination of siloxanes in foodstuffs [46,47].

For example, flame atomic absorption spectrometry was one of the first methods used for the quantitative determination of dimethylpolysiloxane in fats and oils [51] and for the estimation of the amount of dimethylpolysiloxane taken up by food fried in a dimethylpolysiloxane-containing oil [52].

For better selectivity and to increase sensitivity, previous solvent extraction combined with flame atomic absorption spectroscopy was used for the determination of dimethylpolysiloxane in fruit juices and beer [53,54,55].

This ionization methods are very specific for silicon, but they do not allow speciation to distinguish between organic and inorganic bound silicon. For specific PDMS determination in food, a previous extraction with chloroform or other solvents have been described with practically quantitative recoveries (90–95%) and a high precision of determination (1–6%). The main drawbacks of ionization methods are possible interferences with inorganic bound silicon and the potential loss of siloxanes with low molecular mass before atomization.

These problems could be partially overcome using other analytical techniques such as UV and infrared spectroscopy (IR) [56]. Infrared absorption spectroscopy was also one of the first analytical techniques used to determine, after extraction, traces of dimethylpolysiloxanes in vegetable-derived processed foods with silicone antifoams (e.g., pineapple, bread, waffles, hydrolyzed vegetable protein, and frozen vegetables) [57].

However, IR and RAMAN methods, despite being specific for organosiloxanes, are not very sensitive and need massive sample enrichment. Direct analyses in the lower milligrams per kilograms range are not feasible [47].

Hyphenation is a sensitive and useful but slower and more expensive alternative to specific analysis of organic and inorganic silicon. In doing so, size exclusion (SEC) [58] and gas (GC) chromatography [59] has been extensively used to separate silicon species and determine its concentration via several coupled detectors such as ICP, mass (MS) or UV analyzers after separation.

The main difficulty with chromatographic methods is the lack of analytical references for cyclic siloxanes in the mid and high molecular range, which are essential for a reliable quantification. Moreover, the polymeric polydisperse nature of PDMS makes it difficult to use targeted mass spectrometry methods for its quantification.

Therefore, despite the large amount of silicone determinations available, only a few studies have been specifically carried out on the identification of PDMS traces (E900). Most of the applied methods in the literature are not silicone specific or are only for low-molecular-weight silicones from cosmetic products or environmental pollution [37].

## 6. Nuclear Magnetic Resonance for PDMS Analysis in Food

Nuclear magnetic resonance (NMR) is a powerful analytical technique that provides structural information about molecules and their chemical nature. NMR spectroscopy can measure intact biomaterials and foods with little or no sample manipulation, but its low sensitivity usually limits direct measurement of trace compounds in mixtures such as foods.

NMR is based on the excitation of magnetically active nuclei placed in a strong magnetic field with a suitable radiofrequency pulse. From the frequency of the signals emitted by the sample, the analyst can deduce information about the bonding and chemical environment of the atoms in the sample. The values of these resonance frequencies depend on the type of nucleus and the local atomic environment. This latter property produces a fine adjustment of the resonating frequency that provides valuable information about the chemical environment of the studied isotope [60]. NMR is relatively rapid and easy to implement for the analysis of mixtures, metabolic studies, pharmaceutical preparations, natural products, and foods, because it can determine molecular structure, requires a relatively short measuring time, is a non-destructive analysis, requires minimal sample preparation, and can quantify multiple compounds using a single reference as internal calibration [61] or even without it [62].

NMR has the potential to achieve both total silicon determination and molecular identification, mainly in biological fluids and tissues and environmental and petroleum products.

In addition, several nuclei in the structure of PDMS, such as ^1^H, ^13^C, or ^29^Si, could be detected and used to quantify silicones [63]. The carbon and silicon nuclei have been widely reported for NMR determination of polymers of silicon in environmental, biological, or petroleum samples [23], but due to their inherent low sensitivity, these nuclei are not suitable for the detection of traces of PDMS in foods.

However, ^1^H-NMR spectroscopy, after extraction and concentration procedures, is sensitive enough to measure PDMS in food at the level of traces.

In this context, extraction of lipidic compounds from foods may reduce broad signals in the NMR spectra arising from the high overlap of chemical shifts for metabolites and macromolecules and generate narrower and better-resolved NMR resonances that allow for reliable quantification of PDMS [64].

In this way, Helling et al. [65] determined the total content of polysiloxanes in foods migrated from silicone molds using the extracted food’s triglycerides as a standard to quantify. This approach was limited for the fat content of the foodstuffs but gives a limit of detection of 1.9 mg/kg of food with good reproducibility.

Mojsiewicz-Pienkowska et al. [66] overcame the ^1^H-NMR fat matrix problem in edible oils via gentle saponification prior to the NMR measurement. A concentrated sodium hydroxide boiling solution destroyed the fat triglycerides, avoiding PDMS depolymerization. In this work, hexamethyldisiloxane (HDMS) was used as a quantification standard due to the excellent NMR resolution obtained.

**Figure 2:**
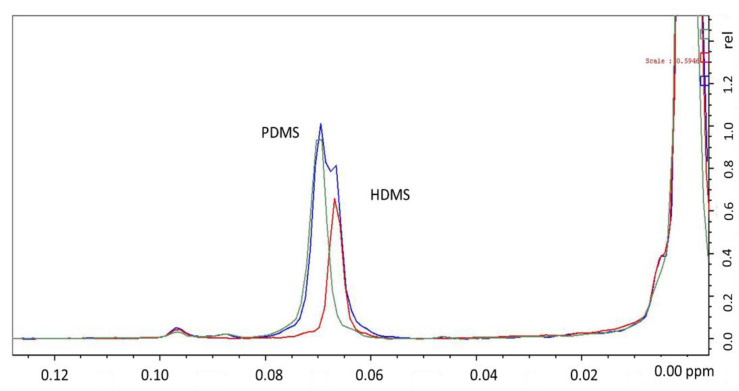
^1^H-NMR spectra of PDMS and HMDS dissolved in deuterated chloroform (CDCl_3_; 500 MHz Bruker NMR) with TMS reference at 0 ppm. Green (PDMS) and red (HDMS) lines show deconvolved compound signals.

In our laboratories (i.e., LJB and COS), we performed saponification following the European standard procedure ECC 2568/91 to obtain an unsaponifiable fraction. Briefly, it consisted of saponifying PDMS- and HMDS-spiked edible oil with ethanolic potassium hydroxide solution under reflux. Unfortunately, PMDS partially hydrolyzed during the process and the NMR resolution between the HMDS and PDMS signals was poor (Figure 2).

## 7. Characteristics of the NMR Signal of PDMS

Therefore, in addition to being useful in food analysis from a global and holistic point of view (“metabolomics” [67,68,69,70,71] and “foodomics” [72,73,74]), ^1^H-NMR could be a suitable technique to quantify E900 and its degradation products and contamination in foods.

Although most of the signals corresponding to the protons of polysiloxanes arise in the narrow region of 0–0.3 ppm, the greater fields of modern spectrometers allow ^1^H-NMR to distinguish between different cyclic, branched, and linear PDMS species.

In addition, lipidic extracts of foods and other biological materials usually do not present NMR signals below 0.5 ppm. Therefore, PDMS and fats, waxes, and other lipidic food extracts could be simultaneously quantified without any interference by overlapping signals.

The dimethylsiloxane moiety represents the main part of the food additive E900, giving in an NMR spectrometer a singlet at 0.07 ppm, which is composed of the six equivalent protons of the [–Si(C**H**_3_)_2_-O–]_n_ unit. This signal is used to directly quantify PDMS, since its area is proportional to the number of protons and, hence, to the amount of additive present. In cyclic dimethylsiloxanes the six equivalent protons of dimethylsiloxy unit are shifted to a lower ppm region, giving a sharp singlet at 0.05 ppm.

Other signals from the PDMS structure can arise. In this way, Helling et al. [14] described the terminal siloxane structures relative to the CHCl_3_ solvent signal referenced to 7.24 ppm in CDCl_3_. Thus, the siloxane terminal units in hydroxyl-terminated linear siloxanes give a signal at 0.16 ppm (HO–Si(C**H**_3_)_2_–O–) and 0.11 ppm (HO–Si(CH_3_)_2_-O–Si(C**H**_3_)_2_–O–). On the other hand, with trimethylsilyl capping, the terminal groups on linear trimethylsilyl terminated siloxanes give a signal at 0.08 ppm of (Si(C**H**_3_)_3_–O–) and 0.03 ppm (Si(CH_3_)_3_–O–Si(C**H**_3_)_2_–O–). This last signal could be overlapped at 0 ppm if too much trimethylsilane (TMS) is added as reference.

The influence of the terminal groups on the chemical shift of other methyl protons is limited to neighboring groups; thus, most of the methyl protons in linear (and cyclic) siloxanes give the abovementioned strong signal at approximately 0.07 ppm, which is only observable if PDMS has a high molecular weight (more than ten repeating units *n* > 10; >0.75 kDa).

**Figure 3:**
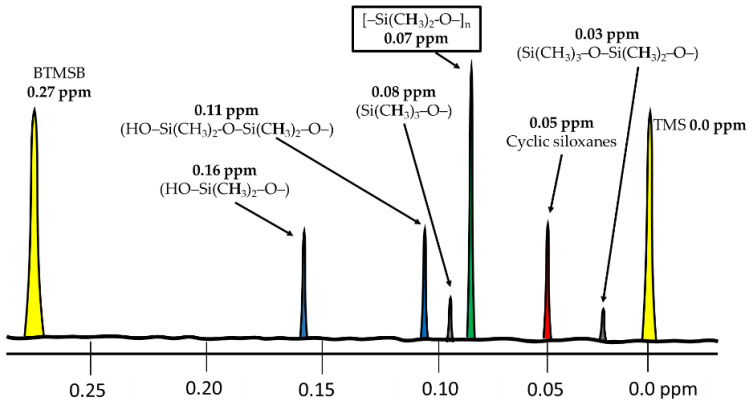
^1^H-NMR chemical shifts of different PDMS moieties and reference substances.

As was stated above, the amount of siloxane was calculated with the sum of the integrations of dimethylsiloxane moieties at 0.07 ppm (Figure 3). By approximation, this calculation method does not include cyclic-, hydroxyl-, and trimethylsilyl-terminated molecules. However, the resulting error (from 3.7% to 0.1%) can be ignored knowing that the range of siloxanes used in the food industry must vary between 0.8 and 30 kDa, and usually ^1^H-NMR signals below 0.06 ppm are not observed.

Despite current legislation [17], if the polymer in the foodstuff were of low molecular weight (*n* < 10; <0.75 kDa) or cyclic, the protons of the terminal units would be observed by ^1^H- NMR, and they would have to be included in the integration.

Moreover, other substances, such as TMS for referencing (δ 0.0 ppm) and HDMS or bis(trimethylsilyl)benzene (BTMSB) (δ 0.27 ppm) for quantification, could be added to the mixture.

## 8. Further Improvements and Use of Internal Standards

It has been proven that ^1^H-NMR is a general and reliable method for the speciation and quantification of PDMS in food. However, extensive extraction and concentration procedures are required depending on the food matrix, the fat content, and the desired level of detection.

In this way, PDMS can be concentrated by extracting it from large amounts of food by using nonpolar solvents. This solvent is then evaporated, and the resulting extract is redissolved to a smaller volume with a suitable NMR solvent such as deuterated chloroform (CDCl_3_). In the literature, the most commonly used solvents to efficiently extract E900 from food are carbon tetrachloride (CCl_4_) [66]; diethyl ether [14], and hexane [59]. As an alternative for restricted carbon tetrachloride (CCl_4)_, we tested chloroform (CHCl_3_). However, hexane showed slightly better recoveries and performance than chloroform and diethyl ether in our experiments (see Supplementary Materials).

Therefore, in the measurement of PDMS in some foods, the variability due to the extraction procedure may be too high; the inherent robustness, precision, and reliability of the NMR technique may not be sufficient to obtain the required analytical specifications.

In order to overcome this problem, the use of an internal standard method could be the best option, since it does not require several independent measurements during one determination process, directly decreasing the determination error and shortening the analysis time. For this reason, we propose a new strategy using an appropriate internal reference.

The added compound must be chemically similar to PDMS but with an NMR spectrum neither overlapped by siloxanes nor food lipidic signals. Moreover, it does not have to react during extraction procedures, it must be easy to manipulate and, preferably cost effective.

In this way, the simultaneous use of various internal standards could improve and correct the analytical variability introduced in the previous PDMS preconcentration and extraction procedures required for the determination in different foods.

**Figure 4:**
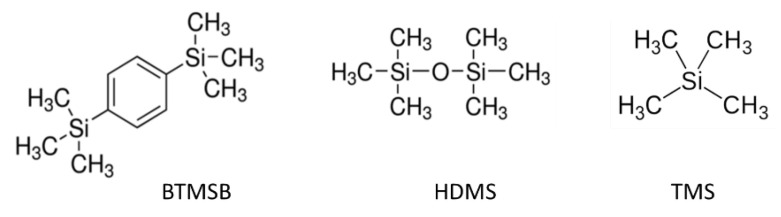
Chemical structure of BTMSB, HMDS, and TMS.

Among the standards used, bis(trimethylsilyl)benzene (BTMSB) (Figure 4) is a good candidate to standardize the quantitative analysis of E900 in any previous pre-concentration procedure in any type of matrix due to the fact of its stability, easy handling, good characterization by NMR, and similarity of physico-chemical properties with PDMS.

BTMSB is a solid compound, lipophilic, and inert to heat, acidic, or basic conditions. It is a known NMR standard with a deuterated form and gives a sharp and isolated ^1^H-NMR signal at 0.27 ppm (Figure 3). Moreover, BTMSB could be added to food before starting any preprocessing or extraction.

The use of BTMSB as an internal reference for the quantification of the E900 food additive fulfils all the requirements and makes different extraction procedures easily generalizable and extensible to various food matrices of different fat contents.

In our laboratories, we performed a validation of the flexibility of this BTMSB internal standard method quantifying the amount of added PDMS in four types of food (surimi fish paste, precooked octopus, canned mussels, and strawberry jam). These quantifications gave analytical parameters comparable to previously published method [66] (Table 1).

Therefore, in our developed method the calibrated range of E900 and the internal standard BTMSB was from 0 to approximately 60 ppm in the NMR tube. As expected, the NMR concentrations responses to BTMSB and PDMS were completely correlated. This calibration curve had a linearity with a coefficient of determination (*r*^2^) of 0.9998. Inter-day precision (0.5 mg/kg, surimi) was determined by analyzing five replicates of five random samples on three different days, and the % RSD was found to be less than 6%. The limit of detection (LOD) based on a calibrated curve was approximately 0.7 mg/kg (S/N > 3), and the limit of quantification (LOQ) was 1.0 mg/kg (S/N > 9) in the NMR tube. Thus, an extraction from five grams of food allowed for the quantification of PDMS until 0.2 mg/kg (see additional experimental details in the Supplementary Material).

**Figure 5:**
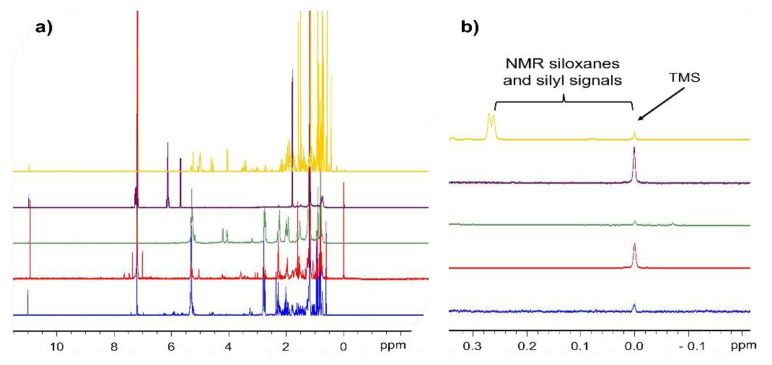
(**a**) Spectra of typical lipidic extracts from different foods: ^1^H NMR spectra from bottom to top: surimi fish paste (blue); precooked octopus (red); mussels (green); strawberry jam (violet); unsaponifiable olive oil extract (yellow) in deuterated chloroform (CDCl_3_). (**b**) Details from 0.3 to −0.2 ppm free of NMR signals from 0.2 to 0.0 ppm (0.0 ppm of TMS reference signal in CDCl_3_) where the PDMS and BSTMS signal appeared.

During PDMS preconcentration for quantification with this method a lipophilic extraction of different foodstuff must be performed. The different ^1^H-NMR food extracts spectra shown lipidic signals of fats and organic compounds such as triglycerides, phospholipids, squalene, waxes, high-molecular-weight alcohols, terpenes, and sterols. Fortunately, all this lipidic substances did not present signals in the region of siloxanes between 0.5 and 0.0 ppm (Figure 5). Thus, clear NMR spectral region of siloxanes without overlapping improved sensitivity, robustness and accuracy of PDMS quantification.

## 9. Conclusions

Silicone-based antifoaming agents (i.e., polysiloxanes or E900) have been shown to be difficult to analyze and quantify due to their polymeric nature and structural variability. Currently, there is no official method for quantifying this additive in food.

In this review, it was shown that NMR is a useful quantitative method for the analysis and differentiation of silicone compounds. A suitable method of PDMS measurement in foods based on a previous preconcentration and a subsequent NMR measurement using calibration and internal standards was described.

It is noteworthy that in this procedure, by using a moderate amount of solvent on foods with a low to moderate fat matrix, the necessary degree of recovery was obtained in a single cycle. Therefore, the use of more solvent, longer extraction times, and/or repetitions would only be required to validate variations in the extraction procedure and/or changes in the solvent to be used. With this quantification method, very small amounts (LOD < 1 ppm) of PDMS can be detected with great accuracy and reproducibility, and with the direct extraction method on fine food preparations with a small amount of hexane (5:1 milliliters of solvent per gram product ratio). TMS could be added to reference spectra at 0.0 ppm, but it is too volatile as a quantification internal standard. Moreover, the addition of BTMSB as an internal standard allows for an improved quantification and compensate for even accidental variability introduced during the extraction procedure. In the case of edible oils, the previous KOH saponification step produces PDMS decomposition; therefore, a gentle procedure that transforms triglycerides without polysiloxane decomposition is needed.

To summarize, we can state that the ^1^H-NMR-BSTMS method is robust enough for assessing the legal compliance of silicone products in food and establishing a reliable exposure for consumers. Further experiments are in progress to apply this method to edible oils.

Based on the results reported here, the suitability of proton NMR for quantitative determination of PDMS in food products has been proven. ^1^H-NMR spectroscopy can be useful for the routine determination of PDMS in food products and other equally complex matrices, such as pharmaceutical products of biological material, when they are combined with a suitable sample preparation procedure. In turn, the variability of the required preparation can be evaluated and corrected using the BTMSB standard, which makes the method presented here extremely robust and easily generalizable, even when the fat matrix size makes it difficult to be detected at low concentrations. Further studies could explore other E900 additive preconcentration techniques and their performance could be easily assessed with the use of BTMSB as internal standard.

## Figures and Tables

**Table 1:** Analytical parameters of the ^1^H-NMR determination of poly(dimethyl)siloxanes in foods using BTMSB as internal standard (recovery rate and relative standard deviation is calculated for surimi fish food). For experimental details see Supplementary Materials.

Analytical Parameter	Value	Units
Calibrated range NMR	0–60	mg/kg (ppm)
Coefficient of determination (*r*^2^)	0.9998	
Extraction recovery	97–103%	(%)
Standard deviation (inter-day)	0.5	mg/kg food (ppm)
Relative standard deviation	5%	(%)
Limit of detection (LOD)	0.7	mg/kg NMR tube (ppm)
Limit of quantification (LOQ)	1.0	mg/kg NMR tube (ppm)

## Supplementary Materials

The following are available online at https://www.mdpi.com/article/10.3390/foods11030297/s1, Supplementary 1: E900 NMR method quantification.
