# Supplementary material for: Considerations on the Analysis of E-900 Food Additive: An NMR Perspective
Source: Foods. 2022 Jan 22;11(3):297. doi: 10.3390/foods11030297 (PMC8833973; doi:10.3390/foods11030297)
Supplement: Supplementary file 1 [file foods-11-00297-s001.zip › foods-1492717-supplementary.pdf]

foods MDPI

Supplementary Materials

CONSIDERATIONS ON THE ANALYSIS OF E-900 FOOD ADDITIVE: AN NMR PERSPECTIVE

Héctor Palacios-Jordan [1], Anna Jané-Brunet [2], Eduard Jané-Brunet [2] , Francesc Puiggròs[3], Nuria Canela-Canela [1] and Miguel A Rodríguez [1,*].

[1] EURECAT, Centre Tecnològic de Catalunya, Centre for Omic Sciences (COS), Joint Unit URV-EURECAT, Unique Scientific and Technical Infrastructures (ICTS), 43204 Reus, Spain; hector.palacios@eurecat.org; nuria.canela@eurecat.org (H.P-J., N.C-C and M.A.R;)
[2] LLUIS JANE BUSQUETS(LJB) Analysis Laboratory S.L. ljblab.com, 08192 Sant Quirze del Vallés , Barcelona, Spain; lluis.jane@ljblab.com (L.J.-B. and A.J.-B.)
[3] EURECAT, Centre Tecnològic de Catalunya, Biotechnology Area, 43204 Reus, Spain; francesc.puiggros@eurecat.org (F.P)
* Correspondence: miguelangel.rodriguez@eurecat.org (M.A.R.).

Tel.: +34-977-775539 (M.A.R.)

Received: 15th November 2021; Accepted: date; Published: date

E900 NMR method quantification.

Experimental Section

1 Description

The goal of the procedure is quantifying the amount of PDMS present in food matrix. A general extraction with non-polar solvent is proposed to isolate the organic fraction composed by fats and lipids together with any present siloxane. The method allows the concentration of PDMS if the amount of lipids in the food is from low to moderate. In the case of high fat content foods such as edible oils, butter or margarine a previous treatment (saponification; skim, etc.) to eliminate fats selectively is needed. An addition of bis(trimethylsilyl)benzene (BTMSB) as internal standard before food processing improves the reproducibility and robustness of method.

2. Methods and Materials

2.1 General

All reagents and solvent used were of analytical grade o higher quality. Hexane, Diethyl Ether and Chloroform were purchased to JT Baker®. Deuterated Chloroform ($CDCl_3$) and tetramethylsilyl (TMS) were from Deutero® GmbH. Potassium Hydroxide (KOH), poly(dimethylsiloxane) (PDMS), hexamethyldisiloxane (HMDS), Bis(trimethylsilyl)benzene (BTMSB) were from a Sigma Aldrich®.

The PDMS used in this work has a molecular weight number (Mn) of 50 *KDa* and a molecular weight mass (Mw) of 95 *KDa* (CAS Number 63148-62-9, viscosity 1,000 *cSt* (25 °C).

NMR 5*mm* O.D tubes were purchased from Cortecnet® in 96 racks for Sample Jet® automation.

2.2 Food sample preparation

The edible oil (olive or sunflower) was prepared according to the European standard EEC 2568/91 to obtain unsaponificable matter. Briefly, the internal standard reference of HDMS, PDMS, BTMSB is added in the desired amount, dried under a stream of nitrogen and 5 *g* of food oil or fat is added. 50 *ml* of 2 *N* potassium hydroxide ethanolic solution is then added to perform hot saponification refluxing for 45 minutes and subsequent biphasic extraction with distilled water (50 *ml*) and ethyl ether (80 *ml*). The upper fraction of ether is collected, the soaps produced are washed with water and rotated until the fraction of unsaponificables keep a constant weight.

The fish, mussels, octopus, jam and any food preparations were homogenized for efficient extraction with hexane (or diethyl ether or chloroform). The procedure was carried out with 1-15 *g* of food grounded with liquid nitrogen in a mortar that was subsequently extracted directly with 3 fractions of 20-40 *ml* of hexane under stirring at room temperature for 10 minutes. The supernatant liquid was obtained by pouring after centrifugation in a 50 *ml* tube to obtain a compact precipitate (4700 *rpm*, 3475 *g*, 15 *min*). Subsequently, the fractions were pooled and evaporated to dryness with rotary evaporation and nitrogen flow to obtain dry lipid extract that was dissolved in 1 *ml* of deuterated chloroform ($CDCl_3$) and measured by NMR.

2.3 *^{1}H-NMR measurement*

The dry lipid extract of the food was completely dissolved in 1 *ml* of deuterated chloroform ($CDCl_3$) with tetramethylsilane (TMS) and 650 µl was transferred to a 5 mm tube. O.D. NMR.

Spectra were measured at 298 *K* on an Avance III 600 spectrometer (Bruker®, Germany) operating at a proton frequency of 600.20 *MHz* using a 5 *mm* gradient PABBO Smart® probe with automatic tuning-matching probe capable of working with proton with an ASTM signal: noise. (950: 1).

Lipophilic samples were measured using a single 90 ° pulse (RD – 90 ° –ACQ; zg from the Bruker® pulse program) with a recycle time (RD) of 20 *s* for adequate quantification. The 90º pulse was calibrated for each sample and ranged between 12.48 and 13.23 *µs*.

The spectral width was 12 *kHz* (20 ppm) and a total of 128 accumulations for the oil samples and 32 accumulations for the references, which were recorded with a resolution of 64 K points for each ^{1}H spectrum. A negative exponential apodization ("line broadening") of 0.3 *Hz* was applied before the calculation of the Fourier transform.

The calculated frequency spectra were phased and corrected manually using TopSpin software (version 3.4, Bruker). They were referenced to the chloroform signal at 7.26 ppm and leaving the siloxane terminal methyl signal of PDMS and HDMS at 0.06 ppm, the BSTM at 0.27 ppm and the TMS at 0.00 ppm.

To corroborate the quantification value, a digital signal (ERETIC-PULCON ref) was introduced at 11 ppm with a calibrated value of 0.6523 *mM* with respect to a standard sample of 2 *mM* of sucrose.

2.4 *Siloxane content quantification*

The extracts obtained from the different foods were mainly constituted by triglycerides and fats in different proportions and in the case of saponification of oils or fats, it consists of their unsaponifiable content of hydrocarbons, ethers, waxes, and sterols. However, all these compounds have their resonance signals in the range of 0.8 to 6 ppm and did not interfere in the quantification between 0.3 to 0.0 ppm of the different standards and silane and siloxane compounds.

PDMS, HMDS and BTMSB were measured in various concentrations in isolation and into food matrices, using the TMS reference standard at 0.0 ppm, to ensure their purity and perform the structural assignment and quantification of the different peaks.

The integration was carried out manually with the TopSpin ® software (version 3.4, Bruker) in the signal at 0.067 ppm for the HDMS, the signal at 0.070 ppm for the PDMS and the signal at 0.27 ppm for the BTMSB referenced to the TMS calibration standard (0.0 ppm, $CDCl_3$). The additional aromatic signal of the BTMSB at 7.27 ppm was not used for quantification but was used for verification of the structure.

The amount of PDMS was calculated based on the following formula:

C_{PDMS}=F [($I_{siloxane}$/6)x($I_{InternalStandar}$/n $_{1H}$) C $_{I.S.}$]/M_{sample} where M_{sample} is the mass of the food (from 5 to 15 *g* food); $I_{siloxane}$ is the intensity (area) of PDMS siloxane groups (0.070 ppm) and $I_{InternalStandar}$ is the intensity (area) of HDMS (0.067 ppm) or BTMSB (0.27 ppm) normalized by their number of protons and multiplied by the concentration C $_{IS}$ (*mg/Kg* or *ppm*) added and F being the extraction recovery factor.

The quantification result of PDMS or E900 extracted from the food can be calculated directly from the previous formula, and this value being already exact for recoveries and quantitative extractions (> 98%; F=1).

To quantify, dimethylsiloxanes represent the main part of the additive E900 giving a singlet at 0.07 ppm, which is formed by the six equivalent protons of the unit [–Si $(CH_3)_2$-O–] $_n$.

To calculate the amount of siloxane in PDMS, the result is calculated by integrating these groups corresponding to the chain and its area is converted to ppm with the high molecular weight standard used for curve calibration. As in general the molecular weight of the additive E900 used in the industry is high, the value of the terminal protons is negligible and the value in ppm is obtained directly regardless of the antifoam polymer used. If the polymer were of low molecular weight (with n <10) the protons of the terminal units would be observed and they would have to be included in the integration.

3 Results

3.1 Sample Foods

For the quantification of PDMS at the trace level, different extractions were carried out on several grams of the finely powdered foods. The foods used initially were mainly composed of an aqueous matrix and consisted of surimi fish preparation, precooked octopus, mussels, and strawberry jam. In all these foods or preparations, the extracted lipid matrix was different and yield a different spectrum in the ^{1}H-NMR measurement, but they showed the region of 0.5-0.0 ppm free of signals.

In the case of vegetable oils and fats, the quantification of PDMS at the trace level, ^{1}H NMR technique was not sensitive enough for direct measurement of PDMS signals up to 30 *ppm* in food. In addition, the lipid matrix precluded direct extraction with lipophilic solvents. Therefore, the oils were subjected to a saponification process that allowed the elimination of the major triglycerides but, unfortunately depolymerized siloxanes in some extend. The NMR spectrum of the unsaponifiables obtained, consisted mainly of hydrocarbons such as squalene, waxes, linear alcohols, terpenes and phytosterols among others. The oil extracts, like previous lipophilic food extracts, showed a region free of NMR signals between 0.5-0.0 ppm. However, the addition of PDMS to these oils, did not provide a quantitative signal at 0.06 ppm, probably due to siloxane depolymerization. Further work is in progress to elaborate a reproducible procedure to quantify PDMS in edible oil matrix.

3.2 Sample PDMS (E900) spiking

Due to the variability of polysiloxane-based antifoamers in the food industry and the lack of suitable reference materials, the sample extraction procedure and PDMS determination by NMR were verified by examining the recovery of a commercial PDMS polymer in spiked samples.

Before spiking PDMS, it was checked that the food samples did not contain any residual previous PDMS (there were no signals within the 0-0.5 ppm range).

In these matrices, PDMS (with CAS number 63148-62-9, *Mw* 95000, *Mn* 50000 ref. 181846 Sigma-Aldrich®) was added before extraction to check the stability of its signal during the extraction procedure. After spiking and solvent extraction, all samples presented the same signal at 0.07 ppm with respect to TMS signal (0.0 ppm, $CDCl_3$).

Bis (trimethylsilyl) benzene BSTMS was also added in known quantity (10 *ppm*) to different foods and oils prior to extraction to check differences in the extraction process and correct variability, giving a signal at 0.27 ppm and another aromatic at 7.27 ppm. The latter is not as useful for integration due to the possibility of interference with phenolic, terpenic and aromatic compounds present into the food.

3.3 Method validation

NMR can quantitatively determine the amount of PDMS, but; despite the quantitative nature of NMR technique, a previous calibration curve of PDMS and BTMSB (from 0 to 60 *ppm*) was elaborated and measured to overcome polymeric nature and to improve quantification of PDMS by NMR in all described experiments.

For E-900 extraction chloroform, diethyl ether and hexane were tested. Extraction recovery was calculated in different food samples (surimi fish paste, precooked octopus, mussels, and strawberry jam) comparing extractions of the PDMS with different solvents. In addition to clean samples with 0 ppm, samples were spiked with three different levels of PDMS added to obtain valid results in a wide range of concentrations (0, 5, 10 and 60 *ppm*).

Chloroform ($CHCl_3$) was used instead of previously reported and currently restricted carbon tetrachloride (CCl_4) to assess E900 recovery extraction. In this way, hexane showed slightly better PDMS recovery than chloroform and diethyl ether even in foods that are more difficult to homogenize such as jams and octopus, probably due to its good tissue penetration.

Anyway, the recovery achieved after extraction was always > 90% for all solvents at all concentrations.

All solvents gave a recovery of PDMS and BTMSB above 90% with only one extraction with a small solvent/food ratio, which was as low as 5 *ml/g* of foodstuff. The similar recovery among different solvents explains the wide range of lipophilic solvents used to extract PDMS in the literature.

For evaluate other analytical parameters, a quantitative extraction with hexane was performed in three fractions (5 *ml* x 3 over 1 g of food) to arrive at recoveries of the added PDMS of 97-103 %.

The recoveries were kept quantitative with a ratio of 15:1 to 5:1 *ml* solvent /*g* of food with hexane.

Interday precision (0.5 mg/kg) was determined by analyzing five replicates of surimi samples from three different days, and the % RSD was found to be < 6%. LOQ = 1.0 *mg/kg* (S/N> 9) and LOD = 0.7 *mg/kg* (S/N> 3) in NMR tube.

4. Discussion

Very small amounts (LOD <1 *ppm*) of PDMS could be detected, with great accuracy and reproducibility; with the direct extraction method on finely powdered fish, mussels and octopus preparations with a small amount of hexane (5: 1 ratio of *ml* solvent/*g* of product). More difficult food matrices, such as jam, could have also a quantitative extraction increasing the amount of solvent (ratio 15: 1 *ml* solvent/*g* jam). In addition, the use of BTMSB as internal standard could improve extraction reproducibility, making NMR a suitable technology for the analysis of PDMS in foods.

However, the edible oil matrices with more fat content give quantification problems, probably due to partial depolymerization and production of foams during the intensive saponification-extraction process. Further work with preparative separations and concentration of PDMS from fat matrices without saponification step is in progress.

5. Conclusions

The suitability of ^{1}H-NMR for quantitative determination of PDMS in food products is established. Therefore ^{1}H-NMR spectroscopy can be useful for the routine determination of PDMS in food products and other equally complex matrices such as, e.g., ex. pharmaceutical products of biological material, when combined with a suitable sample preparation procedure. In turn, the variability of the required preparation can be evaluated and corrected by the use of the BTMSB standard, which makes the method presented here extremely robust and easily generalizable, even when the amount of fat matrix makes its detection difficult at low concentrations.
